# Structural Features and the Anti-Inflammatory Effect of Green Tea Extract-Loaded Liquid Crystalline Systems Intended for Skin Delivery

**DOI:** 10.3390/polym9010030

**Published:** 2017-01-18

**Authors:** Patricia Bento da Silva, Giovana Maria Fioramonti Calixto, João Augusto Oshiro Júnior, Raisa Lana Ávila Bombardelli, Bruno Fonseca-Santos, Camila Fernanda Rodero, Marlus Chorilli

**Affiliations:** School of Pharmaceutical Sciences, São Paulo State University—UNESP, Rodovia Araraquara-Jaú, km. 1, Campus, 14801-903 Araraquara, São Paulo, Brazil; giovana.calixto@gmail.com (G.M.F.C.); joaooshiro@yahoo.com.br (J.A.O.J.); raisabombardelli@yahoo.com.br (R.L.Á.B.); fonsecasantos.bruno@gmail.com (B.F.-S.); camilafrodero@hotmail.com (C.F.R.)

**Keywords:** liquid crystalline systems, water-surfactant-oil based-structures, rheological properties, green tea extract, antioxidant, paw edema

## Abstract

*Camellia sinensis*, which is obtained from green tea extract (GTE), has been widely used in therapy owing to the antioxidant, chemoprotective, and anti-inflammatory activities of its chemical components. However, GTE is an unstable compound, and may undergo reactions that lead to a reduction or loss of its effectiveness and even its degradation. Hence, an attractive approach to overcome this problem to protect the GTE is its incorporation into liquid crystalline systems (LCS) that are drug delivery nanostructured systems with different rheological properties, since LCS have both fluid liquid and crystalline solid properties. Therefore, the aim of this study was to develop and characterize GTE-loaded LCS composed of polyoxypropylene (5) polyoxyethylene (20) cetyl alcohol, avocado oil, and water (F25E, F29E, and F32E) with different rheological properties and to determine their anti-inflammatory efficacy. Polarized light microscopy revealed that the formulations F25, F29, and F32 showed hexagonal, cubic, and lamellar liquid crystalline mesophases, respectively. Rheological studies showed that F32 is a viscous Newtonian liquid, while F25 and F29 are dilatant and pseudoplastic non-Newtonian fluids, respectively. All GTE-loaded LCS behaved as pseudoplastic with thixotropy; furthermore, the presence of GTE increased the *S* values and decreased the *n* values, especially in F29, indicating that this LCS has the most organized structure. Mechanical and bioadhesive properties of GTE-unloaded and -loaded LCS corroborated the rheological data, showing that F29 had the highest mechanical and bioadhesive values. Finally, in vivo inflammation assay revealed that the less elastic and consistent LCS, F25E and F32E presented statistically the same anti-inflammatory activity compared to the positive control, decreasing significantly the paw edema after 4 h; whereas, the most structured and elastic LCS, F29E, strongly limited the potential effects of GTE. Thereby, the development of drug delivery systems with suitable rheological properties may enhance GTE bioavailability, enabling its administration via the skin for the treatment of inflammation.

## 1. Introduction

Green tea extract (GTE) is obtained from *Camellia sinensis* and belongs to the *Theaceae* family. It is native to Southeast Asia, and is currently grown in more than 30 countries around the world. *C. sinensis* has been used often in diets because its major chemical components, flavonoids and catechins present a range of biological activities, such as antioxidant, chemoprotective, anti-inflammatory, and anticarcinogenic activities [1].

The chemical composition of *C. sinensis* has been widely investigated, and it mainly consists of different types of polyphenols, such as flavonoids and catechins. The classes of catechins found in green tea include epicatechin (EC), epigallocatechin (EGC), epicatechin-3-gallate (ECG), and epigallocatechin-3-gallate (EGCG) [2].

The natural active substances in green tea have recently been studied extensively; however, they are unstable compounds, and may undergo reactions that lead to a reduction or loss of their effectiveness and even the degradation of the product. Increasing the stability of the drug is an alternative to increasing its solubility and would still improve the antioxidant and anti-inflammatory activities. Controlled release is achieved by the incorporation of active substances through techniques involving nanotechnology [3,4].

Liquid crystalline systems (LCS) are a type of nanostructured system used to incorporate active substances. Liquid crystals (LCs) have been known since 1889, when Lehmann described an intermediate state in the thermal transformation from solid to liquid. In 1922, Friedel used the term mesomorphic state to define this fourth state of matter; thus, the liquid crystals came to be known as mesomorphic phases or crystalline mesomorphic [5]. LCs are mainly classified as thermotropic and lyotropic, depending on the physico-chemical parameters responsible for the transition phase. In thermotropic systems, the phase transition is dependent on temperature, and, in lyotropic systems, it is dependent on the addition of solvent and the temperature variation. LC lyotropic (LLCs) consist of polar lipids in the presence of a solvent such as water, which hydrates the polar portion of the lipid via hydrogen bonds, whereas the flexible chains of the lipid aggregate into hydrophobic regions rendered, based on the interactions of Van der Waals forces. Among the wide range of possible lyotropic microstructures to be formed with the dispersion of amphiphilic molecules in water, oil, or in the presence of the two components, there are three well-known ways: lamellar (*L*α), hexagonal (*H*_I_ = normal hexagonal and *H*_II_ = reversed hexagonal), and cubic (*q*) phases. The structural arrangement of the lamellar (*L*α), hexagonal (*H*), and cubic (*Q*) phases is obtained by solvation of amphiphilic molecules, which results in different conical or cylindrical geometries. In the lamellar phase, the surfactant molecules form superposed bilayers, while the hexagonal phase organizes itself in cylinders, and the cubic phase appears in a highly viscous three-dimensional structure [6].

LCs are viscous because of their orientation; the rheology helps one to understand the relationships between the viscoelastic and structural properties of these systems [7]. The lamellar phase generally manifests as a viscous liquid, while the hexagonal phase has a gel-like viscosity, and the cubic phase has an extremely high viscosity. The lamellar structure shows a greater similarity with the intercellular skin lipid membrane and is recommended for the development of transdermal delivery systems. Some studies have evaluated the link between the structure and rheological properties of lamellar LCS because rheological measurements offer the possibility of identifying the lamellar phase, thus determining their properties involved in the release of drugs by the transdermal route. However, depending on the concentration and polarity of the aqueous phase solvent, these crystalline structures may undergo variations and structural modifications that may consequently cause changes in the rheological properties of these systems [8]. Thus, the colloidal systems have complex rheological behavior mainly due to particle–particle and particle–solvent interactions, because, in these systems, molecules may bind by chemical bonds (Van der Waals forces) and associate through a mechanical entanglement, hindering the understanding of their rheological properties [9].

The relation between the rheological behavior of various nanostructured systems and their biological activity (anti-inflammatory, antifungal, antibacterial) has been studied by our research group in recent years. By means of rheological tests, our studies have shown that, for instance, the incorporation of drugs and bioadhesive polymers into LCS can enhance their stability due to the elastic nature of these systems. In addition, we also show that thixotropic LCS can increase the biological activity of drugs. These findings reinforced the idea that, through rheological tests, we can screen components to develop safe and effective drug delivery systems [10,11,12].

The aim of this study was to develop LCS stabilized with polyoxypropylene (5) polyoxyethylene (20) cetyl alcohol and correlate the structural features and inflammatory activity of green tea extract (GTE)-loaded nanostructured systems for skin delivery.

## 2. Materials and Methods

### 2.1. Reagents

Polyoxypropylene (5) polyoxyethylene (20) cetyl alcohol was purchased from Volp Indústria Comércio (Osasco, São Paulo, Brazil), lambda carrageenan was obtained from Sigma-Aldrich^®^ (St. Louis, MO, USA), and avocado oil was obtained from MAPRIC^®^ (São Paulo, São Paulo, Brazil). Commercial dermatological cream containing dexamethasone acetate (1 mg/g) was purchased from a local pharmacy (Araraquara, São Paulo, Brazil). Porcine ears were acquired from a local slaughterhouse (Tupã, São Paulo, Brazil).

### 2.2. Methods

#### 2.2.1. Extract

The extract used in all experiments was the green tea glycolic extract (MAPRIC^®^, São Paulo, São Paul, Brazil, Lot PROD 010676).

#### 2.2.2. Construction of Ternary Phase Diagram: Development of Liquid-Crystalline Systems

All the formulations of a phase diagram were put into small glass vials, and polyoxypropylene (5) polyoxyethylene (20) cetyl and avocado oil were weighed in the proportions of 1:9 to 9:1 and titrated up with high-purity water prepared with a Millipore Milli-Q Plus purification system (Merck Millipore Corporation^®^, Darmstadt, Hessen, Germany), and its resistivity was 18.2 MΩ·cm, with the assistance of a pipette to obtain a final amount of 2.0 g. All bottles were heated individually in a water bath at 45.0 °C, shaken vigorously with a glass rod for 5 min, and then sealed. After one day of rest at 30.0 ± 0.5 °C, the bottles were observed against a dark background and classified macroscopically as a phase separation, opaque low-viscosity, opaque high-viscosity, translucent low-viscosity, translucent high-viscosity, or transparent system.

#### 2.2.3. Characterization of Systems

##### Polarized Light Microscopy (PLM)

The three selected formulations were analyzed using PLM before and after loading of GTE. A drop of each formulation was placed on a glass slide, covered with a cover slip, and then examined under polarized light. A Motic^®^ Type 102M Optical Microscope (Motic^®^, Xiamen, Fujian, China) equipped with a digital camera was used to analyze several fields of each sample at room temperature. Photomicrographs were acquired at a magnification of 200×.

##### Flow Rheometry

Continuous flow was analyzed on a controlled-stress DHR-1 rheometer (TA Instruments, New Castle, DE, USA) equipped with parallel plate geometry (20 mm diameter and sample gap of 200 µm) at 32.0 ± 0.1 °C, in triplicate. Samples were carefully applied to the lower plate, ensuring that sample shearing was minimal, and allowed to equilibrate for 1 min prior to analysis. Continual testing was performed using a controlled shear rate procedure in the range from 0 to 100 s^−1^ and back, with each stage lasting 120 s, with an interval of 10 s between the curves. The consistency index and flow index were determined from the power law described in Equation (1) for a quantitative analysis of flow behavior [13]

τ = *k*γη, (1)

where “τ” is shear stress; “γ” is shear rate; “*k*” is consistency index; and “η” is flow index [14].

##### Oscillatory Rheometry

Oscillatory rheometry of the formulations was performed using the same rheometer and parallel plate geometry (8 mm diameter and sample gap of 200 µm) at 32.0 ± 0.1 °C, in triplicate. Samples were carefully applied to the plate as described previously. At first, the stress sweep was performed to determine the viscoelastic region of all formulations. Then, the frequency sweep was performed over a range of 1–10 Hz, which was within the previously determined linear viscoelastic region for all formulations. The storage (*G*’) and loss (*G*”) moduli were recorded. The variation of the storage modulus (*G*’) at low frequencies in a log–log plot of *G*” versus ω followed the power law described in Equation (2), given by:

*G*’ = *S*ω*^n^*, (2)

where *G*’ is the storage modulus, *S* is the formulation strength, ω is the oscillation frequency, and *n* is the viscoelastic exponent.

##### Texture Profile Analysis (TPA)

The mechanical parameters of the formulations were analyzed using a TA-XTplus texture analyzer (Stable Micro Systems, Surrey, UK) via the test TPA. The formulations (8 g) were placed into 50 mL centrifuge tubes (Falcon, BD^®^, Franklin Lakes, NJ, USA) and centrifuged at 4000 rpm for 10 min (Eppendorf 5810R, New York, NY, USA) to remove air bubbles and to smoothen their surfaces. In the TPA mode, the analytical probe (10 mm diameter) descends at a constant speed of 1 mm·s^−1^ and enters the sample up to a predefined depth (10 mm) and returns to the surface at a speed of 0.5 mm·s^−1^. After this first cycle, the machine rests for 5 s, and, subsequently, the second compression starts as the first compression. Hardness, compressibility, adhesiveness, and cohesion were calculated from force–time curves through the Expert Texture Exponent 32 software (version, Stable Micro Systems, Surrey, UK). Three samples of each formulation were analyzed at 25 ± 0.5 °C [15].

##### In Vitro Evaluation of the Bioadhesive Force

The bioadhesive force between the pig ears’ skin and the formulations was assessed via detachment test using a TAXTplus texture analyzer (Stable Micro Systems, Surrey, UK). Fresh porcine ear skin was obtained from a local slaughterhouse and prepared for the test as described by Carvalho et al. [16]. The undamaged skins were removed from the cartilage with a scalpel and a 400-mm thick stratum corneum and epidermis layer was separated from the adipose tissue with a dermatome (Nouvag TCM 300, Goldach, Switzerland). On the day of the experiment, the skin was thawed in physiological saline solution, containing 0.9% (*w*/*v*) NaCl (Merck), at 25 ± 0.5 °C for 30 min; then, its hair was cut with a scissor and it was attached to the lower end of a cylindrical probe (diameter 10 mm) with a rubber ring. The test started with the analytical probe going down at a constant speed (1 mm·s^−1^) up to the surface of the sample. The skin and the sample were kept in contact for 60 s, and no force was applied during this interval. After 60 s, the analytical probe rose at a constant speed (0.5 mm·s^−1^) until the contact between the surfaces was broken. The bioadhesive force of the formulations was measured as the maximum detachment force or the resistance to the withdrawal of the probe, which reflects the bioadhesion characteristic. Three replicates were analyzed at 32.0 ± 0.5 °C [15].

#### 2.2.4. Physico-Chemical Stability Studies

The samples were evaluated for a period of 30 days at room temperature by visual aspects, centrifugation test, pH, and relative density. For visual evaluation, the samples were visually observed for changes such as: color, phase separation, homogeneity, during the one-month period at room temperature. The stability was evaluated by centrifuging 5× *g* of each test sample at 3000 rpm for 30 min. The pH was measured using a pH meter, by using 5% (*w*/*v*) samples diluted in distilled water.

#### 2.2.5. Evaluation of Anti-Inflammatory Effects In Vivo

Male Swiss mice (body weight 25–30 g) were collectively housed in the experimental room for at least 7 days before the experiments. The protocol was approved by the Ethics Committee on the Use of Animals in Research—ECUA School of Pharmaceutical Sciences of Araraquara—UNESP (São Paulo State University) (protocol number 74/2015).

The mice were subdivided into eight groups with seven animals per group: Group I mice were not treated (negative control); Group II received topical dexamethasone (positive control); Group III GTE diluted in water; Group IV—GTE-loaded F25 (F25E); Group V—GTE-loaded F29 (F29E); and Group VI—GTE-loaded F32 (F32E).

Paw edema was induced by an intraplantar injection of 100 µL of 1% (*w*/*w*) λ-carrageenan into the paw of the mice. After 1 h, dexamethasone, GTE or test formulations (100 mg) were applied to the paw. Four hours after the administration of carrageenan, the thickness of the paws were measured (in mm) by using a digital micrometer.

Percent of inhibition of paw edema was also calculated as follows:

(3) $$\%\;Inhibition=\frac{E_{c}-E_{t}}{E_{c}}\times 100,$$

where *E_c_* is the edema of control group; and *E_t_* is the edema of treated group.

The mean and standard deviation of thickness were calculated for each group. One-way analysis of variance was performed, followed by Dunnett’s post hoc test. The difference between the mean edema of treated animals and control group were considered significant at *p* < 0.05.

## 3. Results and Discussion

### 3.1. Construction of Ternary Phase Diagram: Development of Liquid-Crystalline Systems

A nonionic surfactant is used to increase the extension of the microemulsions of regions in phase behavior studies [17]. According to Formariz [18], long hydrocarbon chains are useful for the formation of liquid crystal phases, preventing the possibility of the solvent (usually water) solubilizing the amphiphilic molecule, and result in solutions of dispersed and disordered molecules. Wang and Zhou [19] confirmed that the use of surfactants with a long hydrocarbon chain is responsible for the formation of liquid-crystalline phase systems.

**Figure 1:**
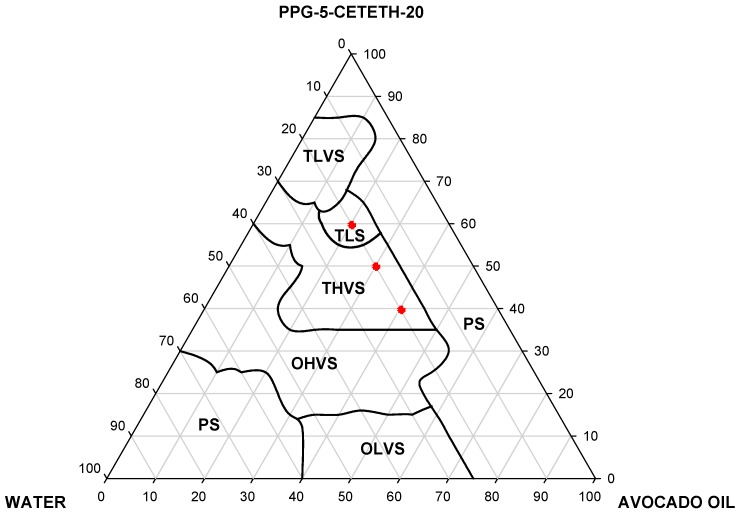
Ternary phase diagram (PPG-5-Ceteth-20, avocado oil and water): opaque low and high-viscosity systems (OLVS and OHVS), translucent low and high-viscosity systems (TLVS and THVS), transparent liquid system (TLS), and phase separation (PS) (the red circle indicates the formulations selected for further experiments).

Thus, the visual assessment of the phase diagram of the test formulations indicated the formation of the following systems: opaque low and high-viscosity systems (OLVS and OHVS), translucent low and high-viscosity systems (TLVS and THVS), transparent liquid system (TLS), and phase separation (PS). When a test tube containing the formulation is inclined at 90°, the meniscus is observed, and the rate of flow of the formulation along the wall of the tube is inversely proportional to its velocity. The phase diagram (PPG-5-Ceteth-20, avocado oil, and water) is shown in Figure 1.

Figure 1 reveals that regardless of proportion of the surfactant several systems with different levels of organization can be obtained. The TLVS region was obtained at a low water (10%), medium oil (70% to 80%) and high surfactant (65% to 85%) content, when the oil was decreased (20% to 60%) and the surfactant remained between 20% and 60%, the region of TLVS was changed to THVS and OHVS. Finally, there were two regions of PS; the first one is up to 40% of oil, above 70% of water and up to 30% of surfactant, while the second region of PS occurred throughout the strip of water, but above 75% of oil and up to 35% of water.

Thus, this diagram shows the interesting ability of these three different components (PPG-5-Ceteth-20, avocado oil and water) to form surfactant systems with varying intensities viscosity. Therefore, three systems were chosen with distinct viscosity to analyze their rheological, bioadhesive, and mechanical properties, as well as to evaluate their anti-inflammatory efficacy. The formulations chosen are indicated in the diagram (Figure 1) and the percentages of each component are shown in the Table 1. These three points were selected in order to maintain the same proportion of water.

### 3.2. Characterization of Systems

#### 3.2.1. Polarized Light Microscopy (PLM)

**Figure 2:**
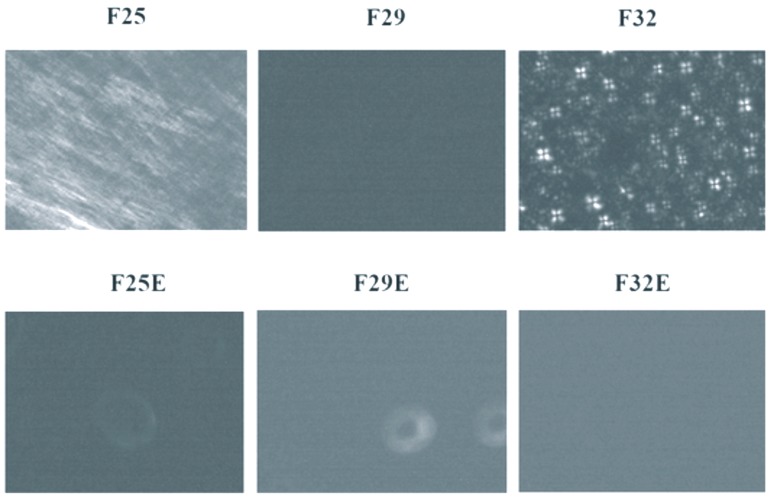
Polarized light microscopy of the selected formulations with and without green tea extract. The formulations F25, F29, and F32 showed hexagonal phase, cubic phase, and lamellar phase, respectively, and all the formulations (F25E, F29E, and F32E) with green tea extract showed cubic phase.

The results from PLM of the selected systems with or without the GTE 0.206 mg/mL are shown in Figure 2 [20].

The results indicated that the selected formulations formed different liquid crystalline mesophases depending on the ratio of the oil and the surfactant, since the amount of aqueous phase was the same for all formulations.

The TLS, namely F32, showed Maltese cross-shaped structures, and, thus, it was classified as lamellar liquid crystalline mesophase. The THVS, F29, is an isotropic formulation because it presented a dark field in the photomicrographs, and was therefore classified as a cubic liquid crystalline mesophase owing to its high viscosity. The other THVS, F25, was classified as hexagonal liquid crystalline mesophase because it showed streaks on the photomicrographs.

All GTE-loaded formulations were classified as cubic liquid crystalline systems because they presented isotropy and high viscosity. Since the extract is a liquid, the hydration of the surfactant may have occurred, increasing the curvature and the volume of the polar region that may have led to the formation of micelles packaged in cubic symmetry [21]. These three points had the same proportion of water but showed different characteristics in PLM and were selected for further analyses (Table 1).

#### 3.2.2. Rheological Studies

##### Flow Rheometry

**Figure 3:**
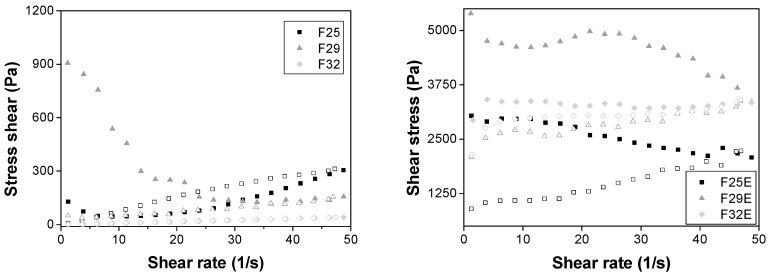
Shear stress versus shear rate of the formulations. Closed symbols represent the up curve and open symbols represent the down curve. Standard deviations have been omitted for clarity; however, in all cases, the coefficient of variation of triplicate analyses was less than 10%. Data were collected at 32.0 ± 0.5 °C.

The flow behavior of a sample under tension, establishing a relationship between the microstructure and macroscopic behaviors of the sample, is studied using this test [22]. Thus, the flow of the formulation from a final package or from the equipment in an industrial process, as well as the spreading of the topical formulation on the skin, is evaluated using continuous rheological analysis [23]. The results are illustrated in Figure 3.

F32 is the only formulation that presented a linear relationship between the shear stress and the shear rate, which is characteristic of a Newtonian fluid, confirmed by flow behavior index (*n* = 1); therefore, its viscosity is constant under stress. This behavior is typical of lamellar mesophase liquid crystalline that presents low viscosity [11].

F25 and F29 are non-Newtonian fluids because they demonstrated a nonlinear relationship between the shear stress and the shear rate. However, F25 is a dilatant fluid (*n* = 1.63) and F29 is pseudoplastic fluid (*n* = 0.49). Both kinds of fluids are useful for topical formulations. The particle volume of F25 expanded, causing a thickening of the formulation after the application of stress and forming a solid paste that can remain in contact with the skin for more time to release the drug. Nevertheless, the opposite behavior was observed in F29 because the flow thinning occurred when a stress was applied, breaking the organized structures, and made it easy to spread over a greater body area. These data show that the type of liquid crystalline mesophase influence the rheological behavior [24].

When the GTE was added in the formulations, all of the formulations behaved as pseudoplastic fluids (*n* < 1) with high consistency index, Table 2, demonstrating that they became more viscous. Furthermore, all extract-loaded formulations presented thixotropy, which is a useful feature since it causes formulations to take longer to return to their organized initial structure, and they can release more drugs during this time [25].

##### Oscillatory Rheology

Oscillatory rheology was performed to investigate the viscoelastic region of the formulations by comparing the elastic modulus (*G*’) and viscous modulus (*G*”). *G*’ is also called storage modulus, representing the energy stored during deformation when tension increases and the energy released when the strain is relaxed. *G*” is the loss modulus since energy that is dissipated in the form of irreversible deformation cannot be stored [26].

**Figure 4:**
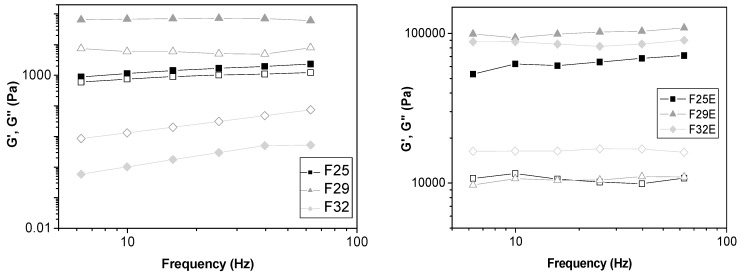
Frequency sweep profile of the storage modulus *G*’ (closed symbols) and loss modulus *G*” (opened symbols) of all formulations.

As observed in Figure 4, F32 was the only formulation that presented *G*” > *G*’, showing to be more viscous than elastic. This finding is typical of lamellar liquid crystalline mesophases due to their low viscosity.

The F29 formulation had the highest value of elastic modulus among the formulations without extract. When the extract was added to the formulations, their elastic moduli increased by approximately 100 times.

Moreover, the data obtained by Equation (2) and shown in Table 3 indicated that the incorporation of extract also increased the *S* values and decreased the *n* values of all formulations. This indicates that the extract-loaded formulation has a more organized and complex structure. Therefore, these data are consistent with TPA data.

Furthermore, this set of results suggest that these liquid crystalline systems composed of Procetyl AWS^®^, avocado oil, water, and GTE are very elastic formulations that can be retained for longer in the skin, thereby increasing the drug absorption time and improving the treatment performance.

#### 3.2.3. Texture Profile Analyses (TPA)

TPA is an analytical technique that may be applied to the mechanical characterization of drug delivery systems by obtaining parameters such as (i) hardness (force required to attain a given deformation); (ii) compressibility (the force per unit time required to deform the sample during the first compression cycle); (iii) adhesiveness (the force per unit time required to detach the sample from the probe during the first compression cycle); and (iv) cohesion (the ratio of the positive force area during the second compression to that during the first compression).

The comparison of such parameters can determine the applicability of the formulation at administration site as well as the therapy outcome, by providing information relating to the ease of removal of a product from a container, the spreadability, and the stability [27]. Moreover, these mechanical characteristics also provide information about the interactions between the systems components [28] that are apropos to developing bioadhesive topical formulations.

**Figure 5:**
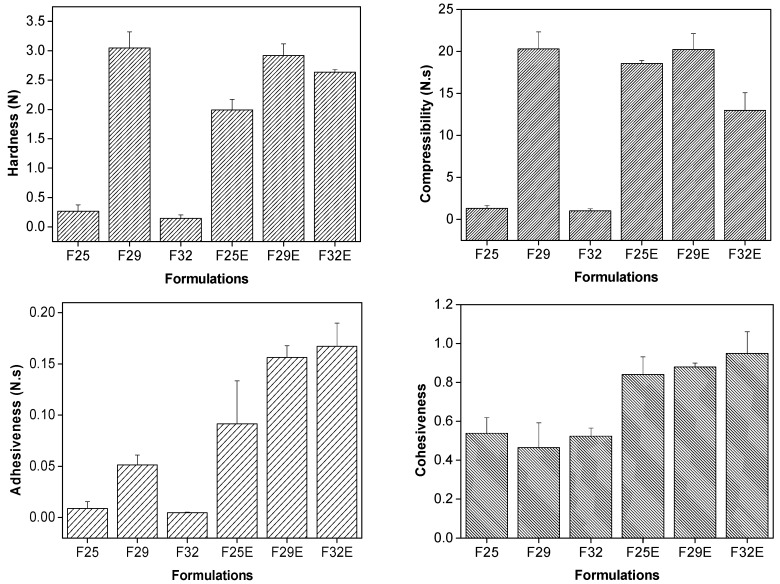
Mechanical properties (hardness, compressibility, adhesiveness, cohesiveness) of unloaded and extract loaded formulations. The data were collected at 25 ± 0.5 °C.

The hardness, compressibility, adhesiveness, and cohesiveness from TPA of the formulations are shown in Figure 5.

F29 formulation exhibited the highest hardness and compressibility values, which may be due to its high viscosity. As observed by PLM, the F29 formulation showed a dark field that indicates cubic crystalline liquid mesophase. The cubic phases consist of normal or reverse micelles packaged in cubic symmetry or that gives viscosity to the system.

All parameters, including adhesiveness, increased when the GTE was added to the formulation. Thus, about four times greater force was needed to separate extract-loaded formulations from the probe. This can relate to the ability of the extract to modify the liquid crystalline arrangement forming more organized structures, which also led to increased viscosity as well as adhesiveness of all formulations [29].

Furthermore, the presence of the extract also increased the cohesiveness of all formulations, which indicates that intermolecular interactions occurred between the components of the formulation and the GTE, forming unbreakable structures with high recovery capacity after stress [29].

#### 3.2.4. In Vitro Evaluation of the Bioadhesive Force

**Figure 6:**
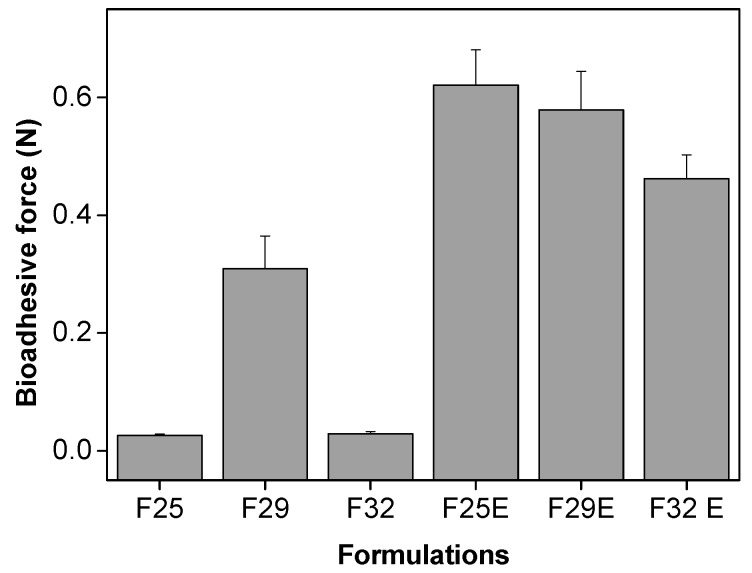
Bioadhesive force (*N*) of the formulations.

The force required to detach the skin of each formulation is shown in Figure 6.

Among the formulations without the incorporation of the extract, F29 was the most bioadhesive. There was a significant increase in the bioadhesion of each formulation upon addition of the extract. These data corroborate the TPA data and indicate that the high viscosity of the cubic liquid crystalline mesophase influences the adhesion of the formulation to the skin surface. Furthermore, the increase in the organizational structure of the formulations caused by the incorporation of the extract resulted in an increase in the bioadhesiveness of all formulations, with values similar to those of polyacrylic acid hydrogels [25]. These data show that these formulations can act as topical bioadhesive formulations.

### 3.3. Physico-Chemical Stability Studies

In a period of one month, no phase separation or changes in color and uniformity of the formulations were observed. The stability after centrifugation was evaluated 24 h post preparation of the formulations. It was observed that the formulations F25 and F29 maintained the same aspect and no phase separation was observed. However, the formulation F32 showed phase separation after 30 min, which may have been due to its rheological properties because F32 presented *G*” > *G*’ and the lowest value of *S*. Thus, this formulation does not form ordered matrices favoring its phase separation [30,31].

The pH was measured 24 h after preparation, and the results are shown in Table 4. The addition of GTE in the formulations caused a decrease in the pH.

### 3.4. Evaluation of Anti-Inflammatory Effects In Vivo

**Figure 7:**
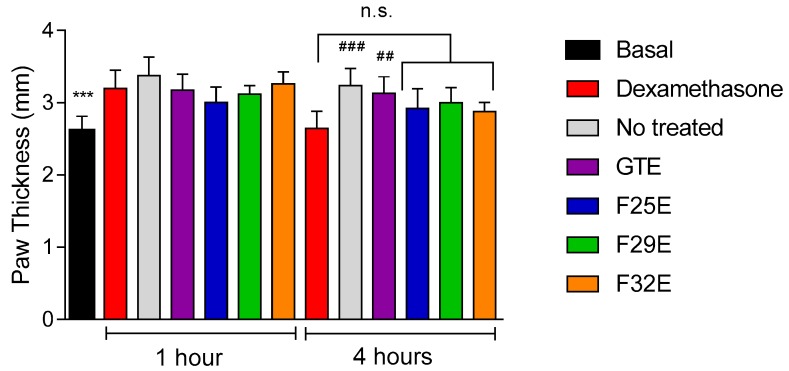
Anti-inflammatory activity of the LCS with green tea extract at 300 mg·g^−1^ (F25E, F29E, and F32E), dexamethasone (positive control—cream containing dexamethasone at 1 mg·g^−1^), and negative control. Notes: the data are represented as mean ± standard deviation of results from five mice. The statistical significance of paw thickness was analyzed using variance analysis via Dunnett’s multiple comparison test; *** *p* ≤ 0.001. vs. Dexamethasone at 1 h of experiment. ## *p* ≤ 0.01 vs. Dexamethasone at 4 h of experiment. ### *p* < 0.001 vs. Dexamethasone at 4 h of experiment. n.s.: no significant difference vs. Dexamethasone at 4 h of experiment.

In vivo anti-inflammatory effects of the formulations are shown in Figure 7. The inflammation in all groups was recorded after 1 h of the experiment. The anti-inflammatory effect of dexamethasone, the positive control, was edema inhibition of 18.3%, after 4 h of treatment. When treated with the nanostructured formulations, F25E, F29E and F32E, the edema inhibition was slightly higher with values of 9.5%, 7.1%, and 11%, respectively. Dunnet’s post hoc test showed that F25E, F29E and F32E significantly decreased the paw edema, similar to that in dexamethasone-treated mice. For unloaded GTE, the inhibition was of 3.4% and was statically different to dexamethasone (*p* ≤ 0.01).

F25E and F32E showed a pronounced anti-inflammatory effect compared to F29E. It is known that the phase behavior and the viscosity of the liquid crystals formulations can affect both the release of the drug and skin permeation, because, in general, formulations with greater viscosity presented a slower drug release.

Hence, the less inhibition of F29E can be due to slow release of the drug in 4 h because this formulation presented the greatest consistency index, *G*’ and *S*, indicating that it is a highly organized network structure formulation

On the other hand, studies show that hexagonal and lamellar liquid crystalline mesophases, such as F25E and F32E, are structurally similar to the skin, and facilitate the permeation of the drug due to favorable skin-penetrating properties. The LC structure is similar to the intracellular lipid structure in the stratum corneum [32].

Furthermore, the less viscous lamellar phases, which favored faster release, seemed to be more adapted to transdermal delivery. Systems involving lamellar phases of monoolein and cineol were good candidates as skin permeation enhancers for propranolol hydrochloride [33]. Studies have shown that hexagonal mesophase formulations can penetrate through the stratum corneum and are promising candidates as transdermal drug delivery systems [34,35,36].

Similarly, curcumin-loaded hexagonal mesophase exhibited an ability to reduce paw edema, possibly owing to the high bioadhesive ability, which was attributed to high contact time with the skin surface, thus promoting the absorption of curcumin [10]. Bioadhesive properties related to liquid crystalline-based systems [14,37,38,39] present important advantages such as long residence times on the application site and reduced product administration frequency [40].

All formulations developed herein have bioadhesive properties that can benefit the anti-inflammatory effects in vivo. Bioadhesive gels have been extensively studied for topical drug delivery with good results. Mepivacaine-loaded hydroxypropyl methylcellulose (HPMC) gel showed prolonged and increased local anesthetic action than the control did, based on the area under the efficacy curve of the rat tail flick test [41]. Another study showed that a formulation of Carbopol 934-based 5-FU (Sigma-Aldrich^®^ (St. Louis, MO, USA)) bioadhesive gel is a better alternative to the traditional cream base for enhanced topical delivery of 5-FU, with reduced skin toxicity [42]. Similar paw edema test showed that poloxamer 407-based bioadhesive gel with 15% penetration enhancer resulted in better permeation and efficacy of ketoprofen [43].

In addition, the liquid crystalline mesophase exhibited good bioadhesive properties on the skin and anti-inflammatory activity, and the latter effect was comparable to that of dexamethasone-containing commercial cream.

Thus, it was concluded that these nanostructured systems are promising drug delivery platforms and can be used as vehicles for topical administration of GTE, as demonstrated by the beneficial biological activity in vivo.

## 4. Conclusions

The mixture of polyoxypropylene (5) polyoxyethylene (20) cetyl alcohol polymer, avocado oil, and purified water could form lamellar, hexagonal and cubic liquid crystalline mesophases; when the GTE was incorporated into the selected formulations, PLM and rheological analyses showed the formation of isotropic cubic liquid crystalline mesophases with high viscosity besides pseudoplastic with thixotropy behavior. Furthermore, GTE-loaded LCSs showed the highest values of both TPA and bioadhesive parameters. Finally, in vivo inflammation assay revealed that the formulations with less elastic rheological properties exhibited statistically the same anti-inflammatory activity as the drug control (dexamethasone) by significantly decreasing the paw edema after 4 h. Therefore, the results above highlight a new and low cost LCS with easy preparation, with suitable rheological properties possibly enhancing the capacity to stabilize and release the GTE for skin administration against inflammation.

## Figures and Tables

**Table 1:** Percentages of components of the formulations.

Formulation	Oleic acid (%)	Avocado oil (%)	Water (%)	O:S ratio
**F25**	40.0	40.0	20.0	1.00
**F29**	50.0	30.0	20.0	0.67
**F32**	60.0	20.0	20.0	0.33

**Table 2:** Consistency index (*K*) and flow behavior index (*n*) for all formulations.

Formulations	*K*	*n*
**F25**	0.52	1.63
**F29**	1168.21	0.49
**F32**	0.98	1.00
**F25E**	3455.21	0.10
**F29E**	5504.35	0.06
**F32E**	3213.89	0.007

**Table 3:** Storage modulus (*G*’), formulation strength (*S*), and viscoelastic exponent (*n*) of all formulations.

Formulations	*G*’	*S*	*n*
**F25**	1567.08	466.32	0.39
**F29**	69,432.90	62,048.60	0.04
**F32**	2.77	0.00	0.00
**F25E**	63,430.82	46,021.71	0.11
**F29E**	101,038.9	86,767.83	0.05
**F32E**	86,293.23	86,651.92	0.001

**Table 4:** pH of the formulations.

Formulations	pH
**F25**	6.67
**F25E**	3.94
**F29**	6.56
**F29E**	4.01
**F32**	6.15
**F32E**	4.20
